# Supplementary material for: Quality of endodontic record-keeping and root canal obturation performed by final year undergraduate dental students: An audit during the COVID-19 pandemic
Source: PLoS One. 2022 Oct 3;17(10):e0275634. doi: 10.1371/journal.pone.0275634 (PMC9529118; doi:10.1371/journal.pone.0275634)
Supplement: Attachment — Submitted filename: Response to Reviewers.docx [file pone.0275634.s001.docx]

**Response to Reviewers**

The authors would like to thank the editor and reviewers for considering the manuscript and their valuable comments. The amendments are made in the revised manuscript.

| **No.** | **Reviewer comment** | **correction** |
| --- | --- | --- |
| Editor | | |
| 1. | Please ensure that your manuscript meets PLOS ONE's style requirements, including those for file naming. | The style requirements were checked. |
| 2. | Please provide additional details regarding participant consent. In the ethics statement in the Methods and online submission information, please ensure that you have specified what type you obtained (for instance, written or verbal, and if verbal, how it was documented and witnessed). If your study included minors, state whether you obtained consent from parents or guardians. If the need for consent was waived by the ethics committee, please include this information | The phrase has been added:  “*No further consent from the patient is needed since written consent forms for patient folder confidentiality have been signed before receiving any dental care*.” |
| 3. | In your Data Availability statement, you have not specified where the minimal data set underlying the results described in your manuscript can be found. | The data availability statement has been addressed. |
| 4. | Please review your reference list to ensure that it is complete and correct. | The reference list has been amended. |
| Reviewer 1 | | |
| 1. | The manuscript is structured, written in a concise manner and in standard English. The topic is interesting, sample size calculation is included in the study, the statistical analysis is appropriate and the results are presented with clarity. I would like to make the following two comments: a) there are no specifics about the status of the two investigators that assessed the PAs. It is important to know if they were undergraduate students, postgraduate students or faculty members. b) It would be preferable to include the p values in the manuscript. | The authors would like to thank the reviewer for the constructive comments.  (a): The words “*senior faculty members*” have been added.  (b). p values were added to Table 3. |
| Reviewer 2 | | |
| 1. | **Introduction**  Please add and explanation on why you assume that record keeping, and treatment quality would be different during the Covid-19 Pandemic than during other periods, as you have defined this as your investigated timeframe. | The authors would like to thank the reviewer for the constructive feedback in improving the manuscript.  Several points were added to the introduction:  “*In addition, strict measurements were implemented to reduce the amount of time and frequency of student-patient contact …… have an impact on students' record-keeping performance*.” |
| 2. | **Materials and Methods**  Endodontic record keeping: How were the 111 patient records selected from all available records from March 2020 to March 2022? E.g., Randomly selected or the first 111? Certain number per year? Please explain.  Please check your tenses throughout the document, it should be past tense, e.g., NA score were excluded instead of will be excluded. | All dental records and dental radiographs of patients who gave their consent and received endodontic treatments from March 2020 to March 2022 were retrieved which has been addressed in the manuscript.  No specific sampling method was applied since all records were used in the study.  The manuscript has been revised and changed the phrases accordingly to past tense. |
| 3. | **Results**  Table 2 Legend: Instead of distribution this should be called documentation of records.  You state that the only acceptable mishaps were no mishaps.  Consequently, your results would be easier to read if you dichotomise into mishaps/no mishaps, instead of acceptable/unacceptable mishaps which could imply that there are such mishaps that are deemed acceptable.  This is also true for Table 3 and the relevant section under “root canal obturation quality”. You state here that the “mishap quality” was “slightly lower” in posterior teeth. Please correct this statement. | The word ‘distribution’ has been changed to ‘documentation’.  The sentences have been revised:  “*Either ‘acceptable’ or ‘unacceptable’ was graded for adaptation, length, and taper. Meanwhile, mishap of root canal filling was evaluated as ‘present’ or ‘absent’. A root filling is defined as satisfactory only when all parameters were graded as acceptable with the absence of mishap*.”  Table 3 has been modified.  The statement has been corrected in the result section. |
| 4. | **Discussion**  Again, you state that “root canal mishaps had the highest acceptability rate”. This conveys the message that mishaps can be acceptable. This should be reworded, for example that mishaps were the variable that occurred with the lowest frequency.  Please discuss why there was such low compliance with post-obturation radiographs, and this is obviously of great importance to ensure that patients don’t leave the clinic before any treatment accidents such as overfilling into the IA canal are ruled out.  Looking forward, did you consider providing students with a documentation template that they could copy/paste into their patient chart to create consistency in record keeping? | The sentence has been rephased.  “*The present results indicated that mishaps were the parameter that occurred with the lowest frequency, while acceptability rates of root canal taper, adaptation, and length were generally high (> 65%)*.”  The reason for low compliance is discussed in the text:  “*One explanation for this could be the time restriction on clinical sessions imposed by the dental school…during the COVID-19 pandemic to establish a correlation with their endodontic record-keeping performance*.”  The sentence has been added:  “*One way to improve this is to provide dental students with a dental documentation template to record dental charts and establish a consistency in dental record-keeping among the students*.” |
| 5. | **Conclusion**  You introduce the category “moderately” satisfactory, while stating in M&M that only if all 4 parameters were graded as acceptable, then the overall case would be satisfactory. Please explain and introduce into M&M or change accordingly. | The conclusion has been rephrased accordingly. |
